# Complete Genome Sequence of Escherichia coli GW-AmxH19, Isolated from Hospital Wastewater in Greifswald, Germany

**DOI:** 10.1128/MRA.00279-20

**Published:** 2020-05-21

**Authors:** Dominik Schneider, Daniela Zühlke, Tabea Petscheleit, Anja Poehlein, Katharina Riedel, Rolf Daniel

**Affiliations:** a Genomic and Applied Microbiology and Göttingen Genomics Laboratory, Institute of Microbiology and Genetics, Georg-August University of Göttingen, Göttingen, Germany; b Department of Microbial Physiology and Molecular Biology, Institute of Microbiology, University of Greifswald, Greifswald, Germany; Loyola University Chicago

## Abstract

The Gram-negative and rod-shaped Escherichia coli strain GW-AmxH19 was isolated from university hospital wastewater in Greifswald, Germany. The genome consists of two replicons, including one circular chromosome (5.04 Mb) and a circular plasmid (126.96 kb). The genome harbors 4,694 protein-coding genes, comprising multidrug resistance and a potential association with urogenital tract infections.

## ANNOUNCEMENT

The strain Escherichia coli GW-AmxH19 is a Gram-negative, mesophilic, and rod-shaped bacterium. It was isolated from 10 ml of saline (0.9% NaCl)-diluted university hospital wastewater in Greifswald, Germany. Members of the genus *Escherichia* are well distributed in wastewater systems all over the world (1, 2). One feature of *Escherichia* spp. is their ability to withstand different antibiotics (3).

E. coli GW-AmxH19 was isolated on LB medium (10 g/liter tryptone, 5 g/liter yeast extract, and 5 g/liter NaCl; Carl Roth, Karlsruhe, Germany) solidified with 15 g/liter agar-agar (Carl Roth) and supplemented with 32 μg/ml amoxicillin (Merck KGaA, Darmstadt, Germany) and grown in LB medium (Carl Roth) at 37°C. Genomic DNA was extracted using the MasterPure complete DNA purification kit (Epicentre, Madison, WI, USA). The isolated DNA was used to generate Illumina shotgun libraries using the Nextera XT DNA sample preparation kit and sequenced on a MiSeq instrument with reagent kit v3 (600 cycles), as recommended by the manufacturer (Illumina, San Diego, CA, USA). For Nanopore sequencing, high-molecular-weight DNA (1.5 μg) was used for library preparation employing the ligation sequencing kit 1D (catalog number SQK-LSK109) and the native barcode expansion kit (EXP-NBD104; barcode 2), as recommended by the manufacturer (Oxford Nanopore Technologies, Oxford, UK). Sequencing was performed using the MinION device Mk1B and the SpotON flow cell R9.4.1, as recommended by the manufacturer (Oxford Nanopore Technologies) for 72 h using MinKNOW software v19.12.2 for sequencing and Guppy v3.4.1 for demultiplexing. Default parameters were used for all software unless otherwise specified. Reads were quality filtered using fastp v0.20.0 (4), and nanopore adapters were additionally trimmed with Porechop v0.2.4 (https://github.com/rrwick/Porechop). Sequencing resulted in 2,427,064 short reads (Illumina) and 213,415 long reads (Nanopore). The bacterial genome assembler Unicycler v0.4.8 (5) was used to perform a *de novo* hybrid assembly in normal mode. This included rotation, which was performed to chromosomal replication initiator protein DnaA (UniProt accession number Q8XBZ3) for the chromosome and RepFIB replication protein A (UniProt accession number Q57154) for the plasmid. The coverages estimated by QualiMap v.2.2.2 (6) using Bowtie 2 v2.3.5.1 (7) and Minimap2 v2.17-r941 (8) were 125-fold (chromosome) and 252-fold (plasmid) for Illumina reads and 308-fold (chromosome) and 550-fold (plasmid) for Nanopore reads. The complete genome consists of one circular chromosome (5,037,322 bp) with an overall G+C content of 50.52% and a circular plasmid (126,964 bp) with a G+C content of 50.49%. The genome annotation was performed with Prokaryotic Genome Annotation Pipeline (PGAP) v4.11 (9). The predicted 4,947 genes included 85 tRNA genes, 22 complete rRNA genes, 6 noncoding RNA (ncRNA) genes, and 4,694 protein-coding genes.

Genome analysis with DeepARG v2 (10) revealed the presence of several genes involved in antibiotic resistance. This comprised genes that confer potential resistance against aminoglycosides, antibacterial free fatty acids, bacitracins, beta-lactams, diaminopyrimidines, fluoroquinolones, fosmidomycins, macrolide and lincosamide antibiotics, glycopeptides, peptide antibiotics, phenicol antibiotics, pleuromutilins, polymyxins, sulfonamides, tetracyclines, and several genes involved in multidrug resistance. The plasmid carried potential resistance against beta-lactam antibiotics and tetracyclines.

### Data availability.

The whole-genome shotgun project of Escherichia coli GW-AmxH19 has been deposited at GenBank under the accession numbers CP048647 (chromosome) and CP048648 (plasmid), with BioProject accession number PRJNA524094 and SRA accession numbers SRR11014407 (Oxford Nanopore reads) and SRR11014408 (Illumina reads).
